# Structural derivatization strategies of natural phenols by semi-synthesis and total-synthesis

**DOI:** 10.1007/s13659-022-00331-6

**Published:** 2022-03-07

**Authors:** Ding Lin, Senze Jiang, Ailian Zhang, Tong Wu, Yongchang Qian, Qingsong Shao

**Affiliations:** 1 State Key Laboratory of Subtropical Silviculture, Zhejiang A & F University, Hangzhou, 311300 China; 2 Zhejiang Provincial Key Laboratory of Resources Protection and Innovation of Traditional Chinese Medicine, Zhejiang A & F University, Hangzhou, 311300 China

**Keywords:** Natural phenols, Structural derivatization, Common fragments, Total synthesis

## Abstract

Structural derivatization of natural products has been a continuing and irreplaceable source of novel drug leads. Natural phenols are a broad category of natural products with wide pharmacological activity and have offered plenty of clinical drugs. However, the structural complexity and wide variety of natural phenols leads to the difficulty of structural derivatization. Skeleton analysis indicated most types of natural phenols can be structured by the combination and extension of three common fragments containing phenol, phenylpropanoid and benzoyl. Based on these fragments, the derivatization strategies of natural phenols were unified and comprehensively analyzed in this review. In addition to classical methods, advanced strategies with high selectivity, efficiency and practicality were emphasized. Total synthesis strategies of typical fragments such as stilbenes, chalcones and flavonoids were also covered and analyzed as the supplementary for supporting the diversity-oriented derivatization of natural phenols.

## Introduction

Natural phenols are a large class of natural products ranging from simple phenol, such as salicylic acid, to complex polyphenol, such as tannins [1]. They widely exist in plants, microbes and foods as diverse forms containing glycosides, ethers, ester and free phenol. Numerous studies have strongly supported the prevention and therapeutic effects of natural phenols against various diseases including cancers [2], cardiovascular [3], neurodegeneration [4, 5], and infection. [6] Dietary phenols, such as tea polyphenols, are also regarded as good antioxidative, anti-inflammatory and neuroprotective agents [7]. Several natural phenols have already been applied in clinic and food industry. Cannabidiol, a non-psychotropic phytocannabinoid of *Cannabis sativa*, has been used as analgesic, antiepileptic and anxiolytic drug [8]. Capsaicin, the famous flavouring agent, was utilized as antiphlogistic in clinic [9]. Natural flavonoids such as eupatilin and genistein were also reported as adjuvant drugs for the treatment of tumors and inflammation [10, 11]. However, most natural phenols were less than satisfactory in efficacy or side effect.

**Fig. 1:**
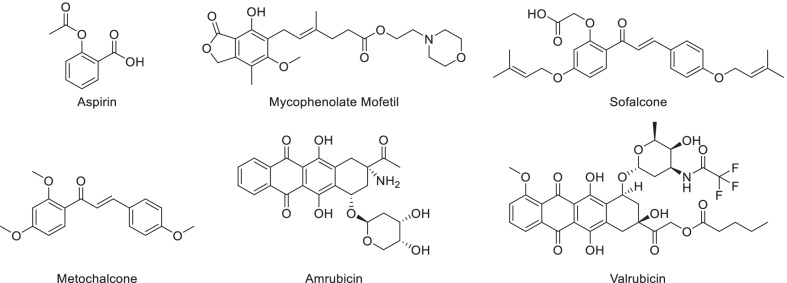
Clinical drugs derived from natural phenols

Structural derivatization is the fundamental way to solve the defects of natural phenols and improve the clinical applicability, and has already been a main approach for drug development [12]. Lots of clinical drugs have been developed by the derivatization of natural phenols (Fig. 1). Aspirin, a nonsteroidal antiinflammatory drug, and mycophenolate mofetil, an immunosuppressor, were structurally derived from natural phenolic acid [13]. The choleretic drug metochalcone and anti-ulcer agent sofalcone were developed through modifying natural chalcone [14]. Synthetic anthracycline antibiotics such as amrubicin and valrubicin were also originated from natural phenols and have been applied in the treatment of tumor [15]. In addition, as it's getting harder to discover novel types of natural phenols on accessible land, it's of growing importance to take full advantage of existing natural phenols and extend the structural types by diversity-oriented derivatization.

Currently there are various derivatization concepts at present such as scaffold hopping [16], structural simplification [17], removal of unnecessary chiral centers [18] and combinatorial chemistry [19], but it’s still hard to formulate derivatization schemes for natural phenols as chemical synthetic feasibility is the precondition. Fortunately, due to some genetic relation of earth's organism, no matter how complex the structures are, natural phenols always consist of common fragments (Fig. 1). Through analyzing the derivatization strategies of common fragments, derivatization schemes for most natural phenols can be easily formulated. Moreover, natural phenols often have multi-reactive sites, multi-chiral centers, complex three-dimensional structures, poor stability and few sources, which set more special requirements for synthetic methods and conditions. The present review not only cover classical derivatization methods, but also advanced strategies with high selectivity, high efficiency and mild condition. For further improving the diversity of derivatization, total synthesis strategies of several representative natural phenols fragments were also discussed, as they could break the limitation of scaffold and source.

## Structural derivatization strategies of natural phenols by semi-synthesis

### Chemical classification and common fragments of natural phenols

**Fig. 2:**
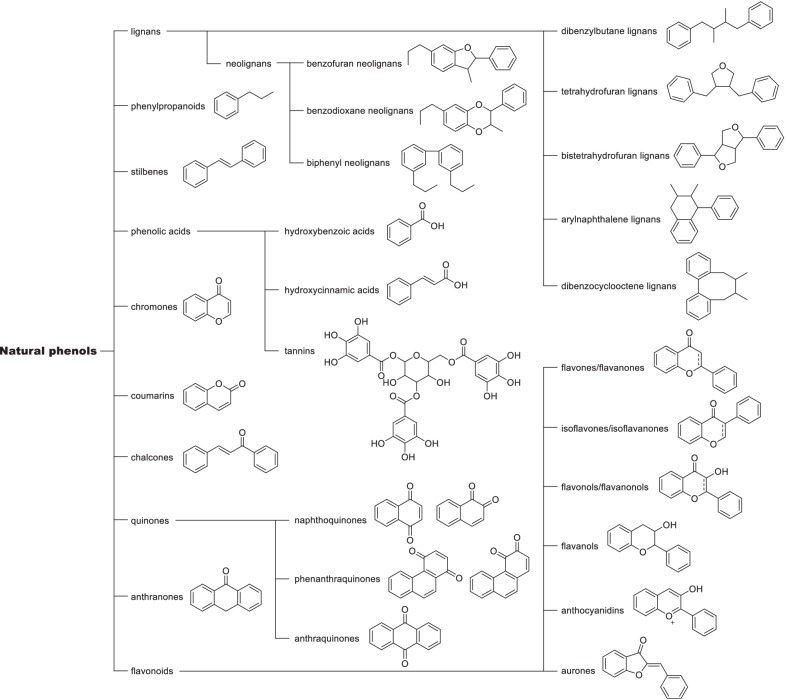
Chemical classification of common natural phenols

Natural phenols were known as a group of natural compounds characterized by at least one phenyl rings and one or more hydroxyl substituents. Common structure types of natural phenols were listed in Fig. 2, ranging from single phenyl compounds such as phenylpropanoids to complicated polyphenols. The big families of lignans and flavonoids were involved. In this review, not only phenols but simple phenolic ethers such as methyl and ethyl phenolic ethers were also included, as they widely exist as the derivatives of natural phenols and shared the same structural modification strategies with natural phenols.

**Fig. 3:**
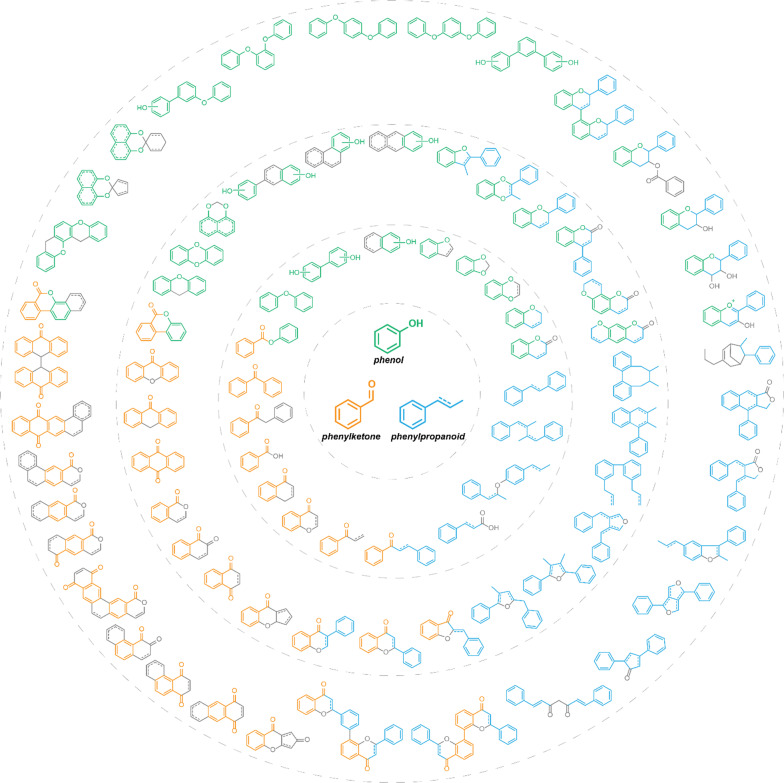
Common fragments of natural phenols

Due to the great variety of natural phenols, it’s unpractical to separately analyze the derivatization strategies for every type of natural phenols. Based on characteristic structures of chemical reactions, structural skeletons of different types of natural phenols were dissected. Three fragments containing phenol, phenylpropanoid and benzoyl were found to be the basic common fragments (Fig. 3). Most types of natural phenols can be structured from the combination and extension of the three common fragments, based on which the derivatization strategies can be unified. For instance, chalcone could be structurally divided into phenol and benzoyl. The derivatization strategies of phenol and benzoyl fragments could also be applied for chalcone. Therefore, the three fragments were regarded as the minimal characteristic fragments and chosen as the template for systematically analyzing the structural derivatization strategies.

### Derivatization strategies of phenol fragments

#### Classical derivatization strategies of phenol fragments

**Scheme 1:**
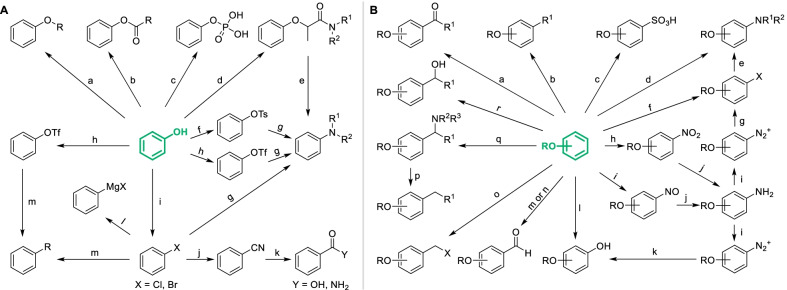
Classical derivatization strategies of phenol fragments. **A** Derivatization of phenolic hydroxyl. Common conditions: **a** ROH, RX, R_2_SO_4_ or R_2_CO_3_ **b** (RCO)_2_O or RCOCl **c** POCl_3_ **d** CH_3_CH(Br)CONR^1^R^2^ **e** base **f** TsCl **g** HNR^1^R^2^, catalyst **h** TfO_2_ or TfCll **i** PX_3_. PX_5_ or POX_3_ **j** cyanide **k** H_2_O **l** Mg, Et_2_O **m** R-M, M = B(OH)_2_, SnBu_3_, etc.; **B** Introduction of various groups onto phenolic ring. Common conditions: **a** R^1^COX/acid catalyst **b** R^1^X/acid catalyst **c** H_2_SO_4_ **d** HNR^1^R^2^/oxidant/catalyst **e** HNR^1^R^2^ **f** halogenating reagents **g** CuX **h** HNO_3_ or t-BuONO **i** NaNO_2_/H^+^ **j** various reductants **k** H_2_O **l** 1) IBX, 2) NaS_2_O_4_ **m** CHCl_3_/NaOH **n** DMF/POCl_3_ **o** HX/HCHO/acid catalyst **p** Pd/C, H_2_ **q** R^1^CHO/HNR^2^R^3^ **r** RCHO/base

**Fig. 4:**
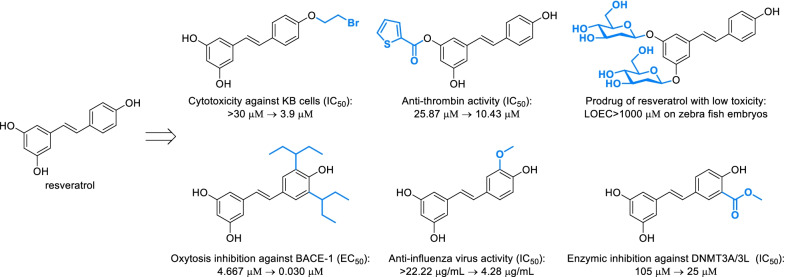
Bioactivity of resveratrol and its analogues derived from modifying phenol fragments

As the basic structure of natural phenols, phenol fragments can be derivatized by modifying the phenolic hydroxyl or introducing specific groups onto the phenolic ring in various classical ways, which were summarized and presented in Scheme 1. Amongst them, electrophilic substitutions of phenolic hydroxyl were the most classical and usual strategies for the derivatization of natural phenols, mainly including acylation, alkylation and phosphorylation [20] (Scheme 1A-a,b,c), which were always applied in designing prodrug to improve the bioavailability or reduce side effect, such as aspirin acylated from salicylic acid for reducing the stimulation on stomach [21], and the glycoside of resveratrol which exhibited quiet low toxicity on zebra fish ombryos (Fig. 3) [22]. Introducing specific groups will also improve the bioactivities by regulating lipid-water partition coefficient or the interaction between compound and biological target. For instance, replacing hydroxyl of resveratrol by bromoethyl and thienylcarbonyl obviously increase the cytotoxicity against KB cells and the anti-thrombin activity respectively (Fig. 4). Similarly, natural phenols can be converted to corresponding sulphonate, but it’s seldom applied in derivatization of natural phenols due to the potential genotoxicity.

Compared to electrophilic substitutions, nucleophilic substitutions of phenolic hydroxyl were less applied in the derivatization of natural phenols which require extreme conditions to break C–O bond due to p–π conjugation, unless there are electron-withdrawing groups on the phenolic ring. Some special approaches enabled to be the condition of nucleophilic substitutions more smoothly and made it applicable for the derivatization of natural phenols. For example, direct ammonolysis of phenolic hydroxyl demand very high temperature which is too dangerous for natural phenols, except natural naphthols which can be ammoniated by Bucherer reaction [23]. Introducing appropriate leaving group such as tosylate (Ts) [24] and triflate (Tf) [25] will reduce the difficulty of cleavage of C–O bond, which always need the participation of metal catalysts (Scheme 1A-f, g, h). In recent years, a metal-free strategy with moderate condition was developed in which arylamine was synthesized by the rearrangement of aryloxy propylamine [26–29] (Scheme 1A-d, e). From the halide intermediate of natural phenols, various groups including cyano, carboxyl and aminoacyl can be introduced through a series of transformations (Scheme 1A-j, k), which have been applied in the derivatization of chalcones [30]. Preparation of Grignard reagent from the halide intermediate was a common strategy to obtain diversiform derivatives (Scheme 1A-l). However, it’s not so applicable for complex polyphenols as it requires sufficient protection of active groups including phenolic hydroxyls. Furthermore, cross-coupling of arylhalide or aryltriflate with organoboron or organometal was also a potential strategy to introduce alkyl or aryl with a great diversity [31, 32] (Scheme 1A-m).

Introducing specific groups onto the phenolic ring may improve the bioactivity of natural phenols. Taking resveratrol for instance, the oxytosis inhibition against β-secretase, anti-influenza virus activity and enzymic inhibition against DNA-methyltransferase 3A/3L increased significantly after introducing certain groups onto the phenolic ring (Fig. 3). As an electronic-rich aromatic ring, not only classical organic reactions including Friedel–Crafts alkylation (Scheme 1B-a), acylation (Scheme 1B-b), sulfonation (Scheme 1B-c), nitration (Scheme 1B-h), halogenation (Scheme 1B-f) and haloalkylation (Scheme 1B-o), but those reactions hardly occurring on aromatic ring such as Reimer-Tiemann reaction (Scheme 1B-m), Vilsmeier-Haack reaction (Scheme 1B-n), Mannich reaction (Scheme 1B-q) and nitrosation (Scheme 1B-i) could also smoothly proceed on phenolic rings, which improve the diversity of derivatization. Similarly to Mannich reaction, natural phenols can be hydroxyalkylated or halomethylated with aldehyde and corresponding reagents (Scheme 1B-o, r). As applied in the derivatization of natural anthraquinones [33], three kinds of alkylated derivatives can be easily converted to each other to obtain a great diversity of molecules. Secondary amine can be directly introduced by various oxidative cross-dehydrogenative coupling reactions or mediately introduced via arylhalide intermediate (Scheme 1B-d,e). Various structures such as hydroxyl and halogen could be further derived from amino through diazotization [34, 35] (Scheme 1B-i, k). The hydroxylation of natural phenols can also be directly activated by 2-Iodylbenzoic acid (IBX) [36], which will selectively generate *ortho*-diphenols (Scheme 1B-k). Nevertheless, it's worth noting that IBX also induces the oxidative demethylation of phenolic ether and the dehydrogenation of acrylophenone, which have been observed in the derivatization of flavonoids [37]. Enzymatic activation was also an available approach for the transformation of phenols to diphenols as similar to biological metabolic processes [38]. Through combining different strategies, more kinds of groups can be introduced onto natural phenols.

#### Selective derivatization strategies of phenol fragments

**Scheme 2:**
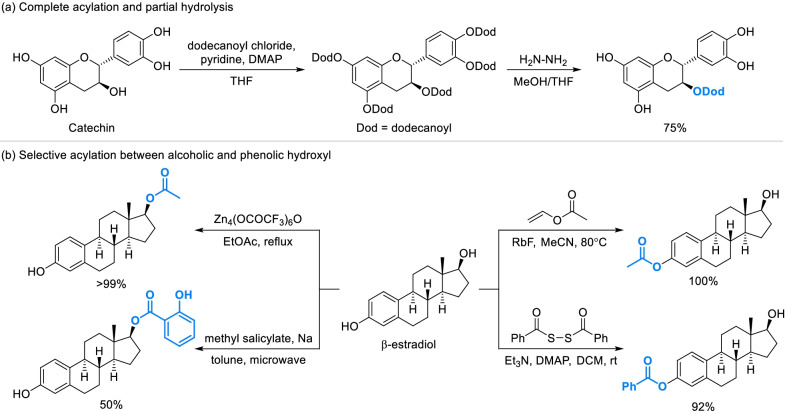
Selective acylation between phenolic and alcoholic hydroxyls

Influenced by complex structure of natural phenols which always contain multi-hydroxyls and rings, classical derivatization strategies probably caused multiple kinds of potential products. In recent years, some selective derivatization strategies of phenol fragments have been applied. As natural phenols always contain both phenolic and alcoholic hydroxyls, it’s necessary to distinguish between two kinds of hydroxyls. One feasible strategy was combination of complete acylation with partial hydrolysis, as alcoholic esters were more stable against moderate hydrolysis [39, 40]. For example, the alcoholic hydroxyl of catechin was selectively substituted by dodecanoyl through 2 steps of esterification and hydrolysis (Scheme 2a). Phenolic hydroxyl and alcoholic hydroxyl can also be directly acylated without affecting each other in the presence of certain additives or by special condition. As shown in Scheme 2b, the alcoholic hydroxyl of β-estradiol was almost completely converted to the alcoholic ester using ethyl acetate as the source of acetyl with the addition of Zn-cluster catalyst [41]. In addition, an investigation of acylation activated by different conditions indicated that microwave will promote the acylation of alcoholic hydroxyl of β-estradiol compared to conventionally heated synthesis (Scheme 2b). Similarly, selective acylation of phenolic hydroxyl can be achieved by special reagents and additive such as rubidium fluoride (RbF) [42] and diacyl disulfide [43] which have significantly accelerated the phenolic acylation of β-estradiol (Scheme 2b).

**Scheme 3:**
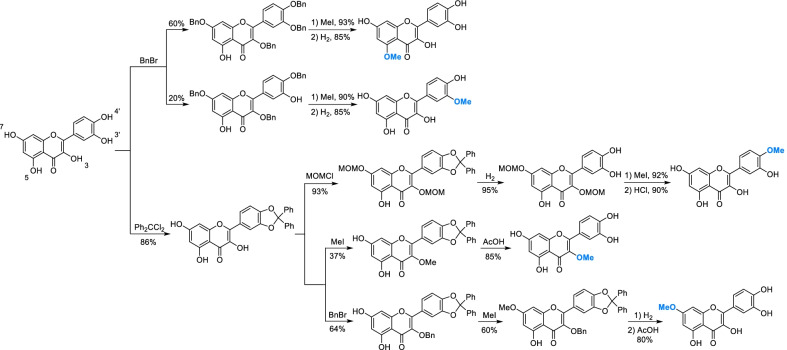
Selective protection/deprotection of hydroxyls of quercetin

For polyphenols, different phenolic hydroxyls can be selectively modified according to the difference of charge distribution and steric effect. Amongst them, selective protection/deprotection is the most common method. As a representative polyphenol, there are five hydroxyls in different chemical environments of quercetin, whose selective protection and deprotection route was summarized and presented in Scheme 3. In general, the priority of electrophilic substitution of five hydroxyls is 4' > 7 > 3 > 3' > 5 [44]. Penta-acetylation of quercetin will transform the substitution order into 7 > 4' > 3 > 5 > 3′ [45, 46]. By controlling the dosage of reagents, protection of hydroxyls can be kept in the situation of trisubstitution and tetrasubstitution, and then 3' -hydroxyl and 5-hydroxyl can be modified separately. Dichlorodiphenylmethane (Ph_2_CCl_2_) could selectively protect two hydroxyls at adjacent position (3' and 4') [47], after which 3-position become more likely attacked by electrophilic reagents such as benzyl bromide and iodomethane.

**Scheme 4:**
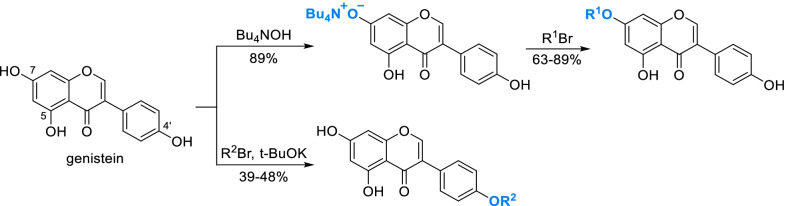
Selective deprotonation of genistein

Selective deprotonation is also a feasible method to selectively modify hydroxyls. Generally, the acidity difference of phenolic hydroxyls will not translate into the selectivity of substitution, while deprotonation will obviously enhance the nucleophilicity of the more acidic phenolic hydroxyl. For instance, 7-OH of genistein was selectively deprotonated by tetrabutylammonium hydroxide (Bu_4_NOH) and then alkylated preferentially [48, 49] (Scheme 4). Otherwise, there will be a substantial percentage of 4'-OH alkylated products [16].

**Scheme 5:**
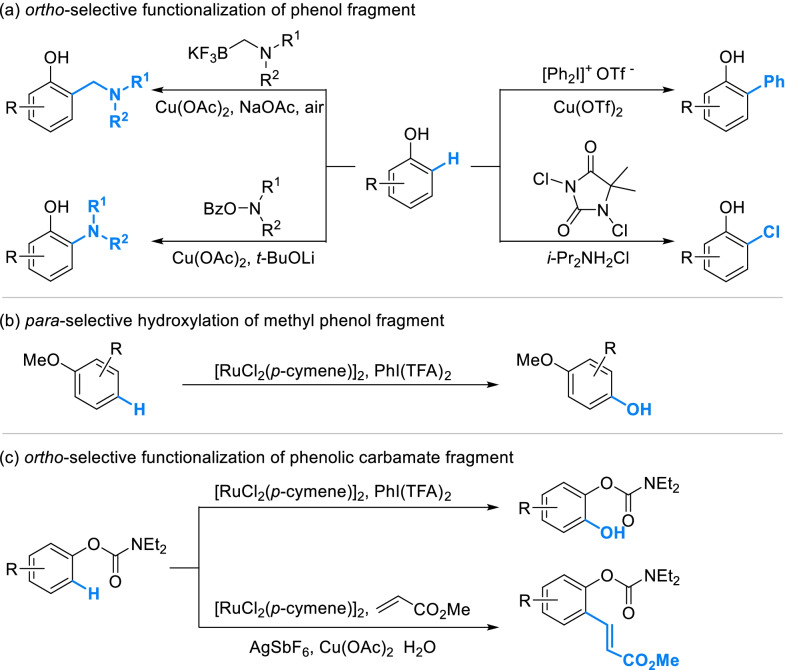
Advanced strategies by regioselective aromatic C–H functionalizations of phenol fragments

Classical strategies for derivatization on phenolic ring (Scheme 1B) showed poor regioselectivity. As activated by phenolic hydroxyl, both the *ortho* and *para* position on phenolic ring are likely attacked by electrophiles. Thus, it’s difficult to obtain derivatives with single and certain component by classical strategies. Recently, some advanced strategies have been developed to regioselectively activate the aromatic C-H bond of phenols, which are being gradually applied in the selective derivatization of natural phenols. As a kind of inexpensive transition-metal catalysts, Cu(II) catalysts were widely researched and applied in the regioselective derivatization of phenols. Amongst them, Cu(OAc)_2_-catalyzed aminomethylation seemed promising in the derivatization of natural phenols for its mild conditions, accessible reagents, high levels of *ortho*-selectivity and group-compatibility [50] (Scheme 5a). Cu(OAc)_2_ could also selectively mediate the *ortho*-amination of phenols with *O*-benzoylhydroxylamines in a moderate yield [51]. Selective *ortho-*arylation of phenols can be achieved in the presence of Cu(OTf)_2_ with diaryliodonium salts, while α-arylation derivative will be produced when the strategy was applied in 2-naphthol [52]. Catalyzed by low concentration of ammonium salt, phenols can be *ortho*-selectively mono-chlorinated with 1,3-dichloro-5,5-dimethylhydantoin (DCDMH) [53], which showed highly practical as all reagents were commercially available. Catalysis of [RuCl_2_(p-cymene)]_2_ was also a common strategy for regioselective derivatization, by which methoxyphenols can be *para*-selectively hydroxylated with the oxidant of PhI(TFA)_2_ [54] (Scheme 5b). In the same condition, phenolic carbamates were selectively hydroxylated on the *ortho*-position (Scheme 5c). [RuCl_2_(p-cymene)]_2_ also catalyzed the selective *ortho*-alkenylation of phenolic carbamates, which was practicable for a large scope of substituted substrates [55]. After hydrolysis, phenolic carbamates can be converted to phenols.

### Derivatization strategies of phenylpropanoid fragments

#### Classical derivatization strategies of phenylpropanoid fragments

**Fig. 5:**
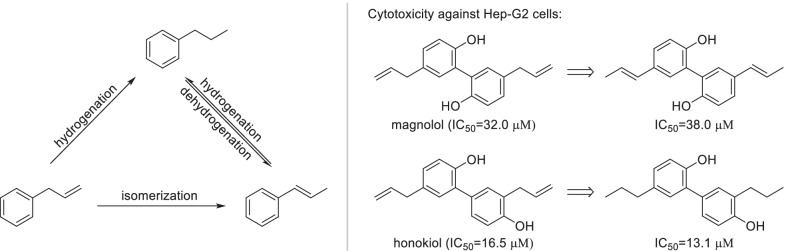
Mutual transformation of phenylpropanoid fragments

Phenylpropanoid fragments, including propyl-benzene, propenyl-benzene and allyl-benzene, are the nuclear structures of various natural phenols including lignans, neolignans, stilbenes and catechins. The three fragments can be transformed to each other by hydrogenation, dehydrogenation or isomerization (Fig. 5). Generally, hydrogenation proceeds in the catalysis of transition-metal such as palladium and nickel using hydrogen gas or ammonium formate as hydrogen source. With the presence of nickel chloride, propenyl-benzene and allyl-benzene fragments can also be hydrogenated by sodium borohydride [56]. Dehydrogenation of propyl-benzenes will give the priority to generating conjugated products such as propenyl-benzenes [57, 58]. Propenyl-benzenes could be transformed by isomerization of allyl-benzenes with miscellaneous methods which have been reviewed in great detail in previous literature [59]. Nevertheless, mutual transformation of phenylpropanoid fragments had relatively little influence on the bioactivity of natural phenols. For instance, both the isomerization of magnolol and the hydrogenation of honokiol made little contribution to improving or reducing the cytotoxicity against Hep-G2 cells, as the IC_50_ values changed little (Fig. 5).

**Scheme 6:**
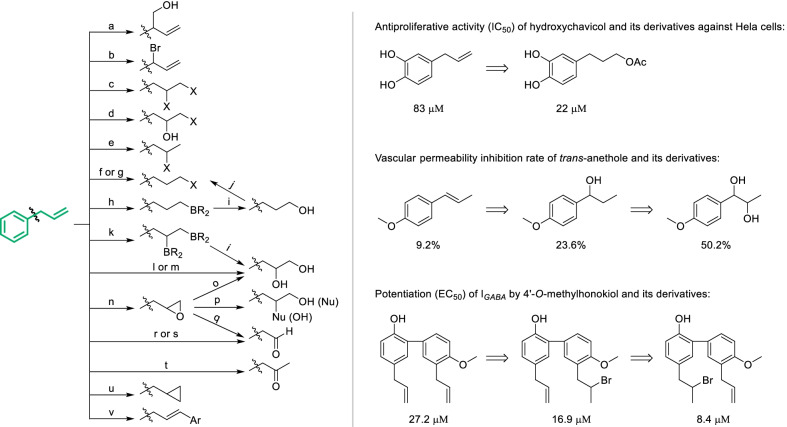
Classical derivatization strategies of phenylpropanoid fragments. Common conditions: **a** (CH_2_O)_n_/*t*BuOK **b** NBS **c** X_2_ or RX/oxidant **d** NXS/NH_4_OAc/H_2_O **e** HX **f** HX/initiator **g** HBr/AcOH **h** R_2_BH **i** H_2_O_2_/base **j** HX **k** B_2_R_2_ **l** OsO_4_ **m** Pd(OAc)_2_/O_2_/base **n** peroxy acid **o** H_2_O **p** nucleophile **q** H_5_IO_6_ or NaIO_4_ **r** OsO_4_/NaIO_4_ **s** O_3_ **t** PdCl_2_/O_2_ **u** CH_2_I_2_/Et_2_Zn/acid **v** ArX/Pd(OAc)_2_/PPh_3_

Compared with mutual transformation of phenylpropanoid fragments, introducing functional groups or specific structures was more likely to promote the bioactivity. As shown in Scheme 6, the pharmacological activities of hydroxychavicol [60], *trans*-anethole [61] and 4'-*O*-methylhonokiol [62] were markedly enhanced by introducing acetoxyl, hydroxyl and bromine respectively into the phenylpropanoid fragments. In addition to those strategies for aromatic ring which have been reviewed in Scheme 1, the side chain of phenylpropanoid fragments offer various possibilities for structural derivatization. Taking allyl-benzene fragment as a template (Scheme 6), it could be converted into an abundance of derivatives by halogenated intermediates. However, halogenation of different conditions will generate diverse types of products, which have been comprehensively reviewed previously [63]. To avoid the halogenation of aromatic hydrogen, the electron density should be reduced by protecting phenolic hydroxyl or introducing electron-withdrawing group. Theoretically, addition of olefin with hypohalous acid could generate halogenated alcohol, but it’s never reported for allyl-benzene fragments. As an alternative approach, allyl-benzenes can be transformed to halogenated alcohol by *N*-halogenated succinimide in the presence of water and catalytic amount of ammonium acetate [64] (Scheme 6d). Addition of allyl-benzene with haloid acid in general condition mainly provided Markovnikov’s products (Scheme 6e), while in the presence of initiator the anti-Markovnikov derivatives can be produced (Scheme 6f). In recent years, the initiator-free anti-Markovnikov hydrobromination has been developed with good scalability and reproducibility, which applied the solution of HBr in acetic acid and required air to be passed through solution in advance [65] (Scheme 6g). Product of single configuration can be obtained by advanced conditions especially rhodium catalytic system [66–68]. Hydroboration with diboride will generate 1,2-bis(boronates) [69] which can be further oxidated to vicinal diol (Scheme 6k). The participation of rhodium [70] and platinum [71] catalysts will lead to enantioselective products. In the presence of osmium tetroxide (OsO_4_), allyl-benzene fragments can be directly oxidized to *cis*-vicinal diol (Scheme 6l), just as the modification of hydroxychavicol [60]. Simple oxidation using molecular oxygen catalyzed by palladium acetate was also developed, but it will furnish a mixture of isomers of vicinal diols [72] (Scheme 6m).

As an important strategy widely applied for natural phenols such as chavicol and honokiol to generate diverse derivatives [73, 74], allyl-benzene fragments can be epoxidized by peroxy acid, after which a great variety of nucleophilic groups including hydroxyl, alkoxyl, aryloxyl, halogen and amino could be introduced [75, 76] (Scheme 6n–p). Affected by periodic acid or periodate, epoxide can be cleaved and transformed to corresponding aldehyde [77, 78] (Scheme 6q). The oxidative cleavage of allyl-benzene fragments can also be directly achieved by OsO_4_ in the presence of excess of NaIO_4_ as co-oxidant (Scheme 6-r), which is easier operated than oxidative cleavage by ozonization [62] (Scheme 6s). In addition, allyl-benzene can be transformed to propiophenone by Wacker-type oxidation by the catalysis of PdCl_2_ [79] (Scheme 6t), meanwhile the internal olefins such as propenyl-benzene will not be influenced. Reaction of allyl-benzene with carbene will generate cyclopropane-type products [80] (Scheme 6u). Furthermore, Heck reaction of allyl-benzene could introduce aryl in the terminal of allyl-benzenes (Scheme 6v), as applied in the derivatization of methyl eugenol [81].

**Scheme 7:**
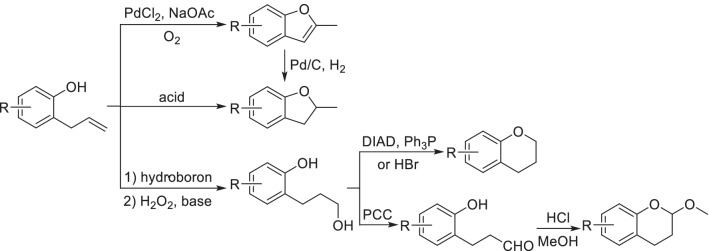
Cyclization strategies of *o*-allyl phenol fragments

**Scheme 8:**
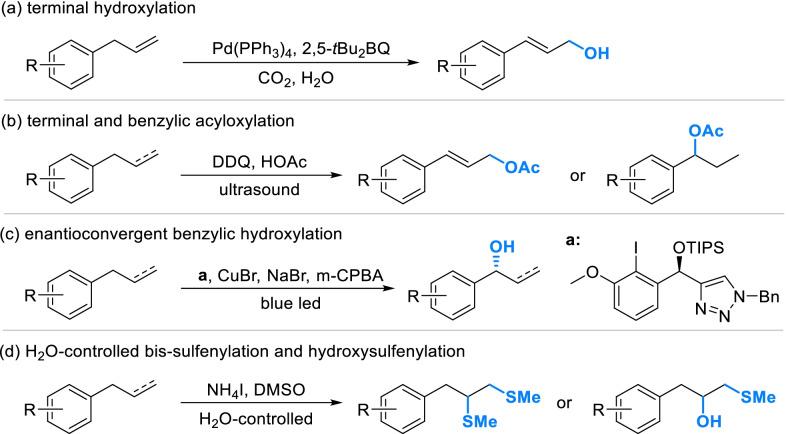
Direct hydroxylation, acyloxylation and sulfenylation of phenylpropanoid fragments

**Scheme 9:**
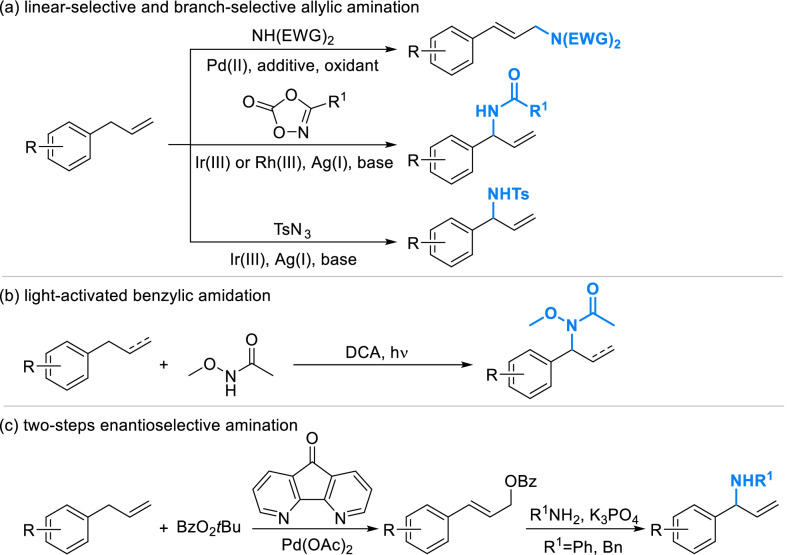
Rapid and selective amination of phenylpropanoid fragments

**Scheme 10:**
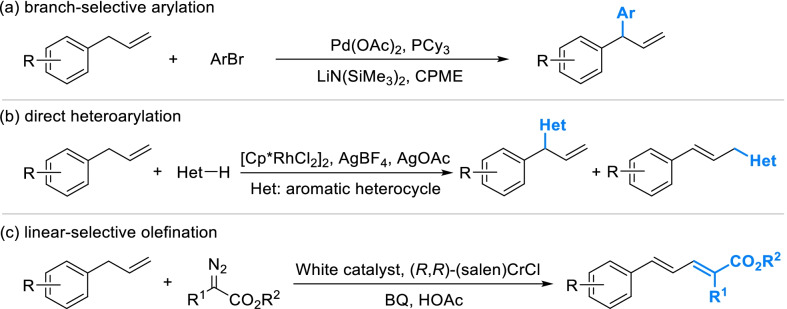
Direct arylation and olefination of allyl-benzene fragments

**Scheme 11:**
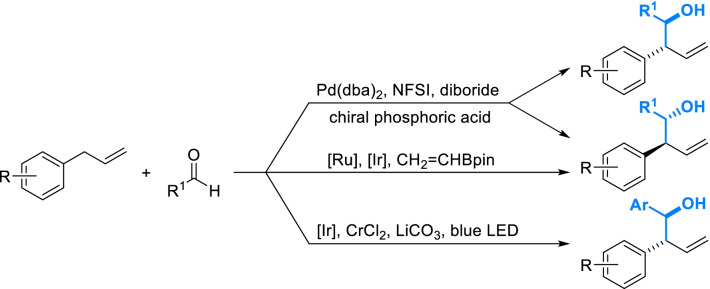
Diastereoselective hydroxyalkylation of allyl-benzene fragments

**Scheme 12:**
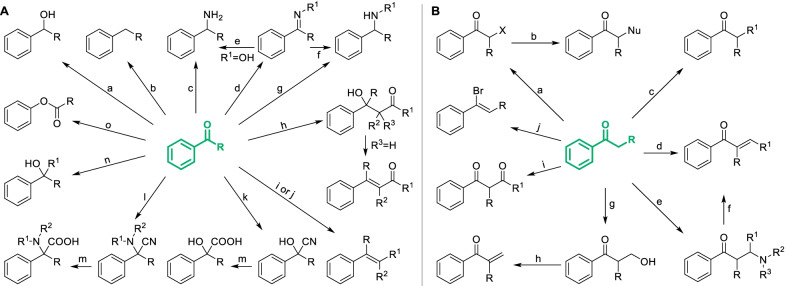
Classical derivatization strategies of phenylketone fragments. **A** Derivatization of carbonyl. Common conditions: **a** NaBH_4_/alcohol **b** Zn-Hg/HCl (Wolff-Kishner reduction) or NH_2_NH_2_/base/ high temperature (Clemmensen reduction) or HS(CH_2_)_3_SH/BF_3_·Et_2_O or NaBH_4_/TFA (Gribble reduction for R = aryl) **c** NH_4_OAc/NaBH_3_CN/alcohol **d** R^1^NH_2_ **e** Pd/H_2_ or Zn/H^+^ **f** NaBH_4_/alcohol **g** R^1^NH_2_/NaBH_4_/Lewis acid **h** R^1^COCH R^2^R^3^ **i** CH_2_R^1^R^2^ (R^1^,R^2^ = elctron withdrawing group) **j** Ph_3_P = CR^1^R^2^ (Wittig reaction) or (OEt)_2_POCHR^1^R^2^ (Wittig–Horner reaction) **k** TMSCN/Lewis acid **l** NHR^1^R^2^/(EtO)_2_POCN **m** H_2_O **n** R^1^MgBr or R^1^Li **o** peroxy acid; **B** Derivatization of acetophenone fragments. Common conditions: **a** halogenating reagents **b** nucleophile **c** R^1^X, base **d** R^1^CHO, base **e** R^1^CHO, NHR^2^R^3^ **f** CH_3_I/base/Δ **g** HCHO, base **h** oxalic acid **i** RCOOEt, base **j** PBr_3_

**Scheme 13:**
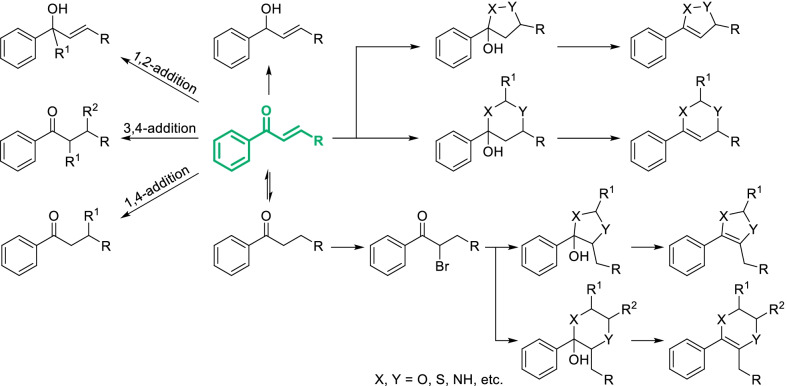
Classical derivatization strategies of acrylophenone fragments

**Scheme 14:**
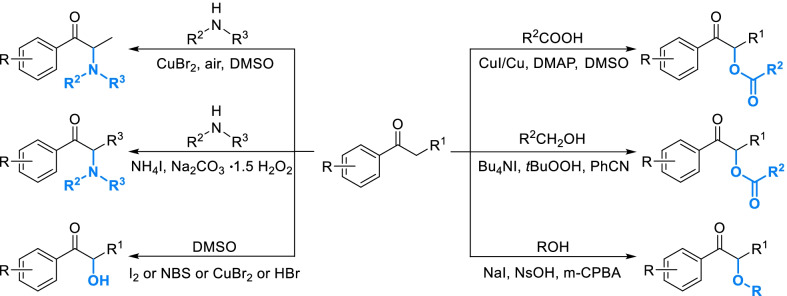
Direct α-functionalization of acetophenone fragment

**Scheme 15:**
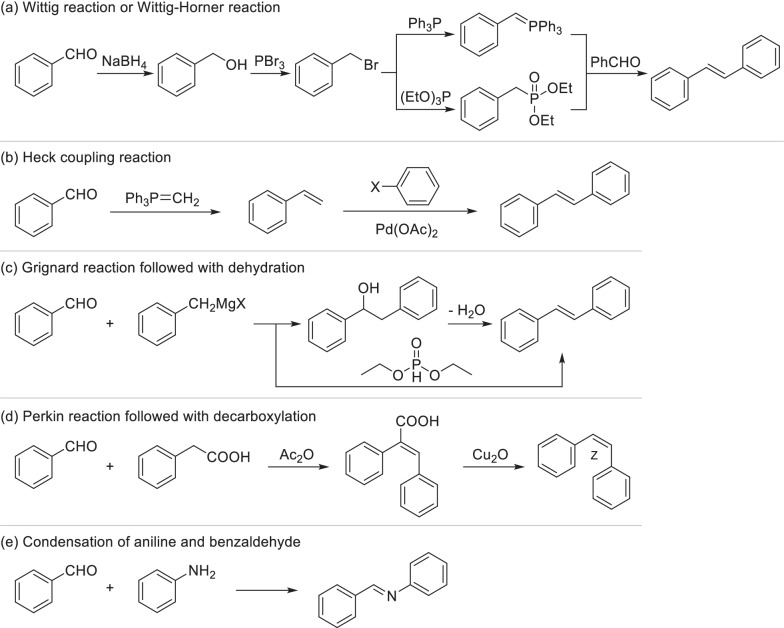
Total synthesis strategies of stilbene fragments

**Scheme 16:**
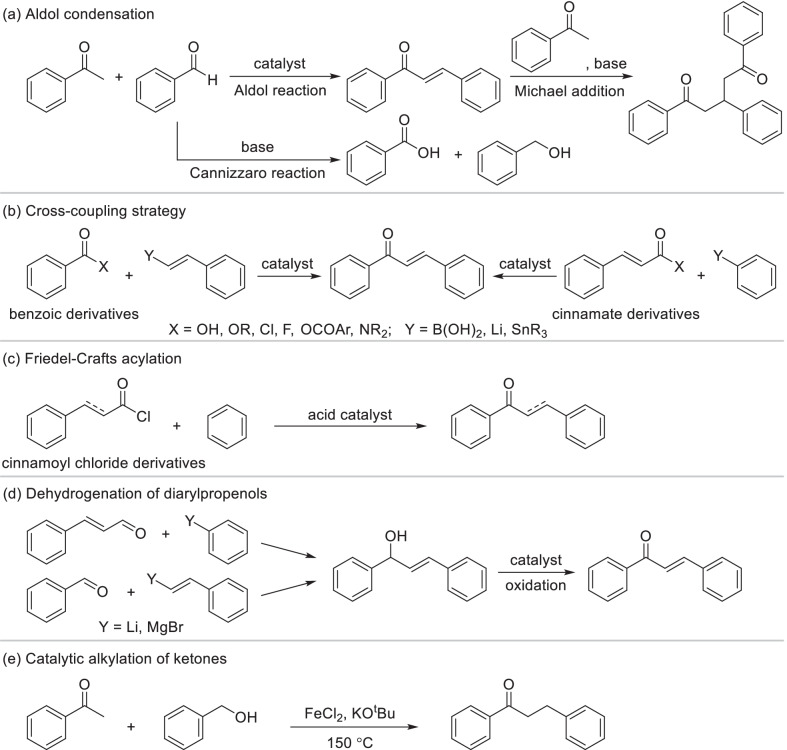
Total-synthesis strategies of chalcone fragments

**Scheme 17:**
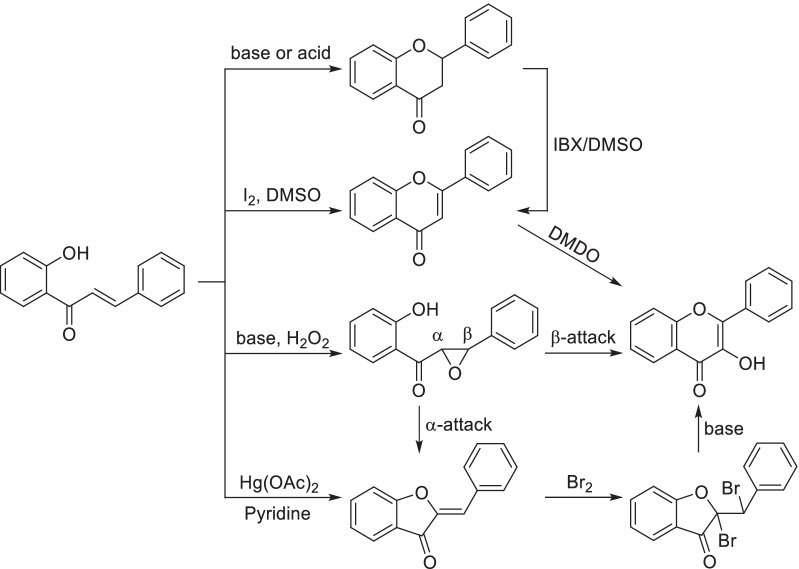
Total-synthetic strategies of flavonoids by cyclization of 2'-hydroxychalcones

**Scheme 18:**
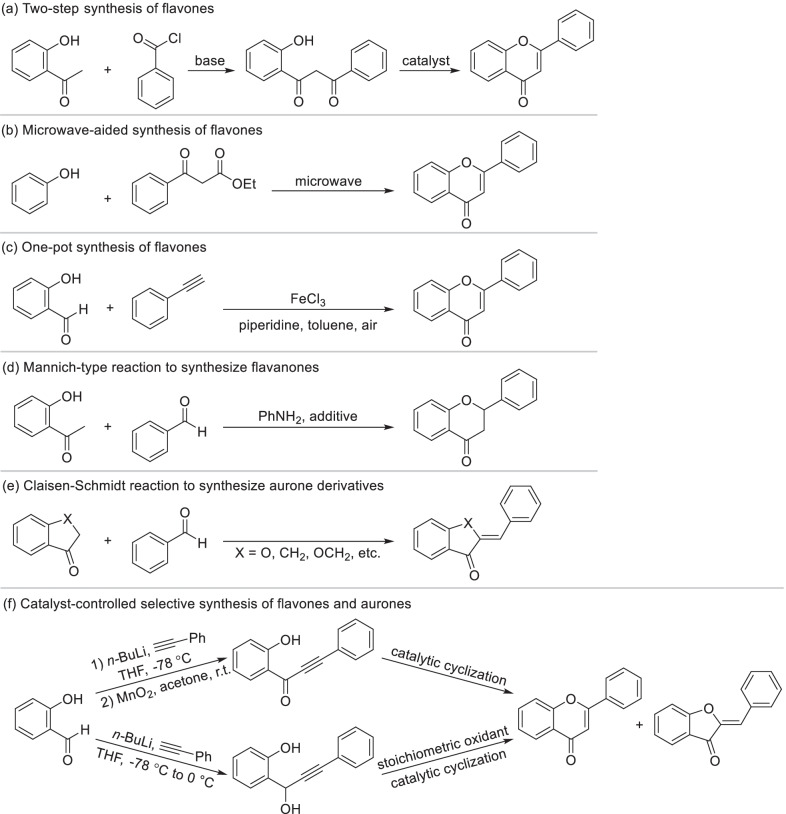
Rapid and novel synthetic strategies of flavones and aurones

**Scheme 19:**
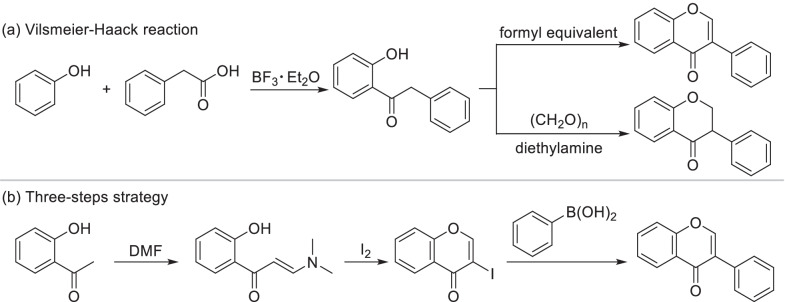
Total-synthetic strategies of isoflavones

**Scheme 20:**
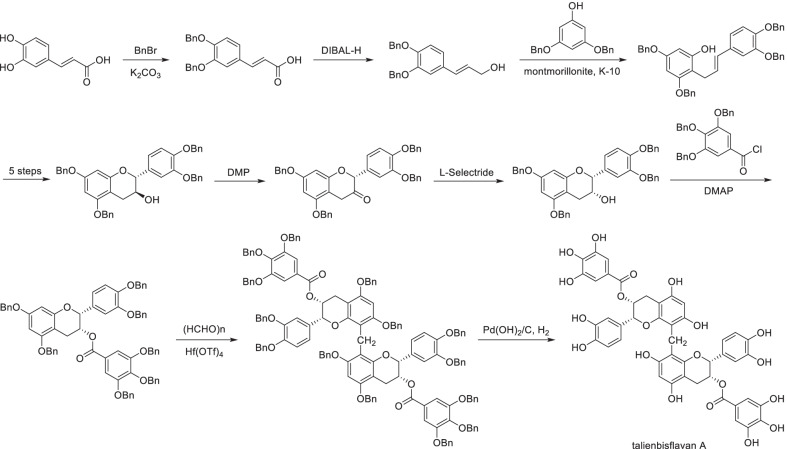
Total-synthesis of talienbisflavan A

**Scheme 21:**
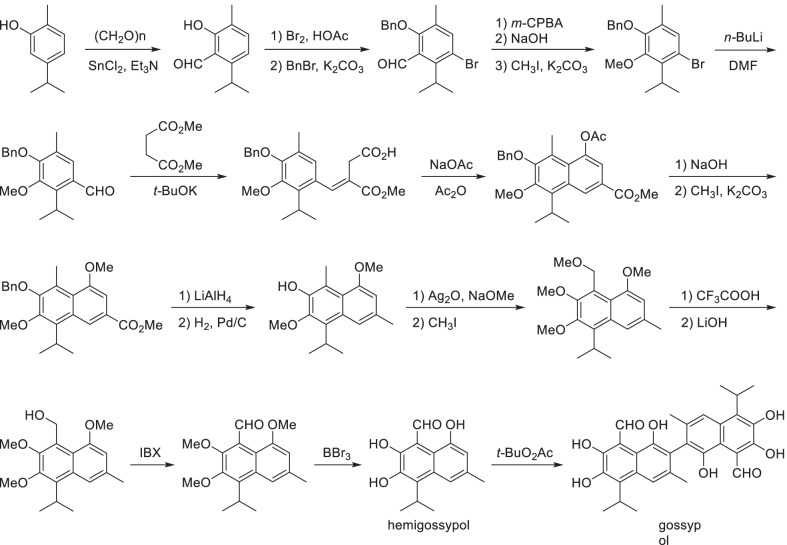
Total-synthesis of gossypol

For expanding the diversity of derivatives, the skeleton of natural phenols can be reconstructed by ring formation. As a common fragment of natural phenols, *o-*allyl phenol can be oxidized and cyclized to give methyl benzofuran by the catalysis of PdCl_2_, which is known as Wacker-type oxidative cyclization [82] (Scheme 7). In the presence of copper salts, oxidative cyclization could undergo without molecular oxygen [83]. By intramolecular acid catalyzed cyclization, *o-*allyl phenol can be converted into methyl dihydrobenzofuran [84], which can also be obtained by the hydrogenation of methyl benzofuran [85]. However, acid-catalyzed polymerization might be observed if there are multi-olefin groups in the structure of natural phenols. Through hydroboration and intramolecular dehydration in the presence of diisopropyl azodicarboxylate (DIAD) and triphenylphosphine, *o*-allyl phenols will be transformed into dihydrochromenes [86], which can also be obtained by the reflux of phenylpropanol intermediate with hydrobromic acid [87]. Oxidation of phenylpropanol intermediate by pyridinium chlorochromate (PCC) followed with condensation will generate 2-alkoxydihydrochromenes [86].

**Fig. 6:**
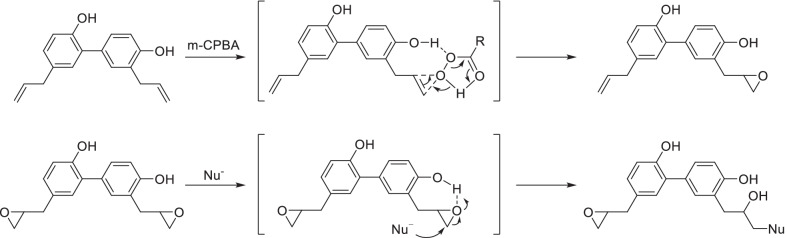
*Ortho*-effect of phenolic hydroxyl

In addition, a special strategy has been developed for selective derivatization of side chain taking the advantage of the *ortho*-effect of phenolic hydroxyl, as the side chain in the *ortho* position of phenolic hydroxyl has higher priority in some reactions due to the intramolecular and intermolecular interaction. It’s reported that the epoxidation of allyl of honokiol were obviously promoted by the intermolecular hydrogen bonding between m-CPBA and *ortho*-hydroxy, and the epoxide in the *ortho*-position of phenolic hydroxyl was more likely to be attacked by nucleophilic groups [74] (Fig. 6).

#### Rapid and selective derivatization strategies of phenylpropanoid fragments

The introduction of functional groups into phenylpropanoid fragments always required several steps by classical strategies, which caused low efficiency, poor selectivity. It’s also a great waste for precious natural phenols. Recently, advanced strategies have been developed with high efficiency and selectivity. Allyl-benzenes can be directly oxidized into primary allylic alcohols by the catalysis of [Pd(PPh_3_)_4_] with H_2_O, CO_2_ and 2,5-di-*tert*-butyl-1,4-benzoquinone (2,5-*t*Bu_2_BQ) as reagents (Scheme 8a). With ultrasonic treatment, allyl-benzenes can be simply transformed to terminal ester by 2,3-dicyano-5,6-dichlorobenzoquinone (DDQ), while for propyl-benzene acyloxylation preferentially occurred in the benzyl (Scheme 8b). In the presence of triazole-substituted chiral iodoarene, allyl-benzenes or propyl-benzenes can be converted into chiral benzyl alcohols by an enantioconvergent hydroxylation (Scheme 8c). Recently, a NH_4_I-promoted direct sulfenylation of was accomplished in a special way controlled by water. A series of β-hydroxysulfide were produced from allyl-benzenes in mixed solution of DMSO and water, while bismethylsulfanes can be obtained in anhydrous system (Scheme 8d).

Amino was an important group to improve bioactivity and realize diversity-oriented derivatization. As the classical strategy, it will take several steps to introduce amino onto phenylpropanoid fragments through the halogenated intermediate. Direct amination of allyl-benzenes can be activated by transition-metal catalysis or photocatalysis. The catalysis systems of Pd(II) promoted the coupling of allyl-benzenes and secondary amine with electron withdrawing groups (EWG) such as acyl and tosyl, which always generated linear allylic amines [88–91] (Scheme 9a). A series of branch-selective allylic amidation using dioxazolones were also developed, which proceeded in a mild condition catalyzed by Ir(III) or Rh(III) and extended to a wide scope of amide [92–94]. Branch-selective sulfamidation can be promoted in similar condition using tosyl azides (TsN_3_) as the nitrogen source [95]. A novel visible-light-catalyzed reaction could directly introduce *N*-methoxyl amide into benzyl position employing 9,10-dicyanoanthracene (DCA) as an absorbing photoredox catalyst (Scheme 9d), but the universality was unknown as there was only few cases related to phenylpropanoid fragments in this research [96]. Allyl-benzenes can also be aminated mediately by benzoate with high enantioselectivity [97] (Scheme 9e). Although it underwent two steps, the conditions and reagents were relatively available. A wide scope of nucleophiles was reported in this work, but only phenylamine and benzylamine were successfully applied for allyl-benzenes.

Direct introducing arylation and olefination onto phenylpropanoid fragments has seldom been reported up to now, as catalytic formation of inactive C–C bond remains a challenge. At present, a cross-coupling reaction in the presence of Pd(OAc)_2_ and tricyclohexyl phosphine (PCy_3_) has been established to selectively introduce aryls into the branch of allyl-benzenes (Scheme 10a), which was reported to be appropriate for a wide scope of aryl groups [98]. Catalyzed by [Cp*RhCl_2_]_2_, allyl-benzene fragments can be activated to give heteroaromatic products with poor selectivity [99] (Scheme 10b). Activated by White catalyst and (salen)CrCl, allyl-benzene fragments can be directly transformed to conjugated polyene derivatives employing α-diazo esters with good stereoselectivities [100] (Scheme 10c).

Direct hydroxyalkylation of allyl-benzene fragments can be enabled by basic catalyst, but it showed poor stereoselectivity [101]. In recent years, a series of transition metal-catalyzed strategies have been developed to keep product in a single enantiomer. Amongst them, oxidative borylation seemed to be the most promising strategy with moderate condition and low cost (Scheme 11). Hydroxyalkyl derivatives of different configurations can be obtained by employing different diborides and chiral phosphoric acids as ligands [102, 103]. Allyl-benzene fragments can also be diastereoselectively hydroxyalkylated catalyzed by ruthenium and iridium catalysts through a cross-metathesis/ isomerization/allylboration sequence [104]. Besides, a hydroxyalkylation enabled by the combination of photoredox, iridium and chromium catalysis could exclusively produce 1-hydroxyl-1-aryl-methyl derivative as a single diastereomer from *cis*/*trans* mixture of electron-rich allyl-benzenes [105].

### Derivatization strategies of phenylketone fragments

#### Classical derivatization strategies of phenylketone fragments

**Fig. 7:**
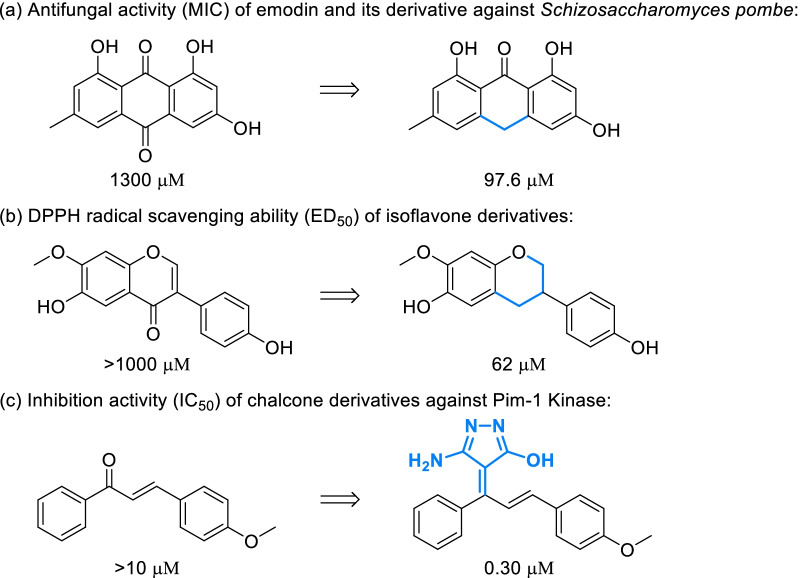
Bioactivity of natural phenols and their analogues derived from modifying phenylketone fragments

As the basic scaffold of chalcones, flavonoids and polyketides, phenylketone is also a very common fragment of natural phenols, which always exist in the form of diarylketone, acetophenone, propiophenone, acrylophenone, benzoic acid and ester. Modification of phenylketone fragments can create an enormous variety of different derivatives and even change the skeleton of natural phenols, also greatly affecting the bioactivity. As a famous natural phenol with excellent pharmacological effects, emodin was converted into a mono-ketone derivative which showed significantly stronger antifungal activity toward *Schizosaccharomyces pombe* [106] (Fig. 7a). Similar phenomena has been observed in the research of isoflavone derivatives (Fig. 7b), whose radical scavenging ability was greatly enhanced after reduction of phenylketone fragments [107]. Introducing specific groups onto phenylketone fragments may also influenced the biological activity, such as the addition of pyrazole onto chalcone derivative obviously improving the inhibition against Pim-1 Kinase [108] (Fig. 7c). More classical strategies were presented in Scheme 12 for designing diversity-oriented derivatization based on phenylketone fragments.

As the classical strategies, phenylketone fragments offered two kinds of possibilities for structural derivatization: the carbonyl and aliphatic side chains. The carbonyl of phenylketone can be transformed into various structures by classical reactions (Scheme 12A). Amongst them, reduction of carbonyl into methylene can be achieved by several methods (Scheme 12A-b). Classical strategies mainly including Wolff-Kishner reduction and Clemmensen reduction were appropriate for phenylketone with good stability. The approach through dithiane intermediate could convert carbonyl into methylene in a mild condition, which was widely applied in the synthesis and modification of natural products [109]. Carbonyl can also be mildly reduced using triethylsilane and acid catalyst such as boron trifluoride etherate (BF_3_·Et_2_O). Diarylketone can be easily converted into diarylmethane by Gribble reduction [110]. For anthracene-9,10-diones such as emodin, the reduction of carbonyls can be controlled to mono-reduction by SnCl_2_ in mixed acid, and the carbonyl close to hydroxyl can be protected probably due to the intramolecular hydrogen bond [106]. Carbonyl can also be reduced to primary amine in the presence of NH_4_OAc and NaBH_3_CN [111] (Scheme 12A-c) or via the intermediate of oxime [112, 113] (Scheme 12A-e). The condensation and hydrogenation can be combined in one-pot by the catalysis of Lewis acid such as CeCl_3_ [114] and titanium isopropoxide [115] (Scheme 12A-g). As a common strategy, phenylketone fragments can be translated to hydroxyphenylacetone or α,β-unsaturated ketone by Aldol reaction (Scheme 12A-h). Similarly, nucleophilic addition followed by elimination of phenylketone could provide styrene derivatives with elctron-withdrawing groups (Scheme 12A-i). Through Wittig-type reactions, more kinds of styrene derivatives can be obtained (Scheme 12A-j). By an addition reaction with cyanotrimethylsilane (TMSCN), phenylketone can be converted into α-cyanoalcohol derivatives (Scheme 12A-k). In the presence of amine and cyanide such as diethyl cyanophosphate (PO(OEt)_2_CN) and acetone cyanohydrin, phenylketone can also be translated to α-cyanoamines, which was known as Strecker reaction (Scheme 12A-l). α-Cyanoalcohols and α-cyanoamines can be further hydrolyzed and generate to α-hydroxyacid and α- aminoacid, respectively (Scheme 12A-m). Nucleophilic addition with organometallic compounds such as organolithium and Grignard reagent will convert phenylketone into α-alkyl or α-aryl alcohol derivatives (Scheme 12A-n). Moreover, phenylketone fragments can be transformed to corresponding phenylester by peroxy acid which was known as Baeyer–Villiger oxidation [116] (Scheme 12A-o).

For acetophenone fragments, there were many options of derivatization on the aliphatic side chains. The α-hydrogen of carbonyl can be replaced by halogen (Scheme 12B-a), which can be further substituted by electrophile such as amine (Scheme 12B-b). Acetophenone fragments could also react as nucleophile with halohydrocarbon or carbonyl compound to lengthen the carbon chain (Scheme 12B-c,d). The reaction with carbonyl compound was known as Adol-type condensation. Mannich reaction is a classical strategy converting acetophenone fragments to α-aminomethyl products [117] (Scheme 12B-e), which can be further transformed into α,β-unsaturated ketone by Hofmann elimination (Scheme 12B-f). α-Hydroxymethyl product can be obtained by Tollens condensation (Scheme 12B-g), and eliminated in the presence of oxalic acid (Scheme 12B-h). Acetophenone fragments can also be α-acylated by carboxylic esters which is known as Claisen condensation, while formyl can be introduced by formate [118] (Scheme 12B-i). In addition, acetophenone fragments with stable enol form can be brominated in the presence of phosphorus tribromide (Scheme 12B-j), which can be further substituted by nucleophiles [119].

Derivatization strategies of acrylophenone fragments were generally similar to those of phenylketones and phenylpropanoids. However, catalytic hydrogenation of acrylophenone will preferentially occur at C–C double bond, while the carbonyl could be selectively hydrogenated by sodium borohydride with the addition of CeCl_3_ which is known as Luche reduction [120] (Scheme 13). Dehydrogenation of propiophenone fragments always employ 2,3-dicyano-5,6-dichlorobenzoquinone (DDQ) as oxidant [121]. Recently, an efficient, economic and general Pd(OAc)_2_-catalyzed dehydrogenation was developed using molecular oxygen as the oxidant [122], offering a new option for this transformation. 1,2-addition and 3,4-addition of acrylophenone fragments were also conducted in similar way with those of phenylketone and allylbenzene fragments which has been respectively reviewed in Schemes 12 and 6. As a particular strategy, 1,4-addition could introduce various groups including hydroxyl, alkoxyl, amino and alkyl with electron-withdrawing groups onto β-carbon of acrylophenone fragments [123]. Acrylophenone can also be transformed to five- or six-membered heterocycles by addition of reagents with double nucleophilic groups such as hydrazine, hydroxylamine, urea and thiourea [124]. Guiding by the same strategy, propiophenone fragments can be converted into various types of heterocycles including imidazole, thiazole and piperazine [125].

#### Rapid derivatization strategies of acetophenone fragment

α-Functionalization of acetophenone fragment is an important way to produce a great diversity of derivatives. However, introduction of most functional groups such as hydroxyl and amino by traditional strategies required multiple steps, which caused low yield and efficiency. In recent years, lots of one-step strategies have been developed. Some of them seemed to be practicable for the derivatization of natural phenols (Scheme 14). Direct α-amination was completed by the catalysis of copper bromide in an atmosphere of air, but the substrates were restricted to secondary amines [126]. A transition-metal-free α-amination strategy employed ammonium iodide as catalyst and sodium percarbonate as oxidant, and the scope of amine was extended to primary amine [127]. There are a lot of research on direct α-hydroxylation of acetophenone fragment. Amongst them, the most practical strategy is employing dimethyl sulfoxide as oxidant and catalytic amount of halogenous reagents such as iodine and *N*-bromosuccinimide [128, 129]. Single isomer of α-hydroxyl acetophenone derivatives could be generated in the presence of chiral bimetallic palladium(II) complex [130]. Direct α-acetoxylation of acetophenone fragment can be realized by different strategies. A copper-catalyzed system seemed to be an accessible and broad-spectrum way with the substrates ranging from simple carboxylic acid to cinnamic acid derivatives [131]. Catalyzed by Bu_4_NI, α-acetoxylation can be accomplished from alcohols [132]. Direct α-alkoxylation can be activated by catalytic amount of sodium iodide with *m*-CPBA as the oxidant and *para*-nitrobenzenesulfonic acid (NsOH) as the additive [133].

## Structural derivatization strategies of natural phenols by total-synthesis

Semi-synthesis strategies will rapidly expand the molecular library from natural phenols, but hardly change the skeleton, and are unaccessible for those natural phenols with rare sources. Total-synthesis is a significant supplement for diversity-oriented derivatization, as it will not be limited by the natural skeleton and sources. Take resveratrol as an example, total-synthesis strategy could produce its monohydroxy, dihydroxy and trans derivatives, which are unachievable for semi-synthesis. Herein, the classical and advanced total-synthesis strategies of typical natural phenol fragments were reviewed, which include stilbenes, chalcones, flavonoids and complex natural phenols.

### Total-synthesis strategies of stilbene derivatives

Stilbenes are a class of compounds with a nucleus of diphenylethene or its polymer, which are mostly contained in the xylem of plants. When plants are subject to pests or external stimuli, the content of stilbenes in the stimulated parts will increase significantly. The most common strategies to artificially synthesize stilbene fragments are Wittig reaction [134] and Wittig–Horner reaction [135] of phosphorus ylide with various substituted benzaldehyde (Scheme 15a). Phosphorus ylide can be produced by the reaction of benzyl bromide, which was provided from corresponding benzaldehyde through reduction and bromination, with triphenylphosphine or triethyl phosphite, respectively. Common synthetic methods of stilbenes also include Heck coupling reaction, in which stilbenes were produced by substituted vinylbenzene and phenylhalide in the presence of palladium catalyst [136] (Scheme 15b). Substituted vinylbenzene can be furnished by Wittig reaction from corresponding benzaldehyde. As a highly stereoselective reaction, Heck coupling could give the product of single *trans*-configuration. Grignard reaction followed with dehydration was another common approach to obtain stilbenes [137, 138] (Scheme 15c). In the presence of diethyl phosphite, benzyl magnesium halide and benzaldehyde could directly convert to stilbene [139]. As a kind of natural isomers, cis-stilbenes can be obtained by a two-step synthesis (Scheme 15d). At first, Perkin reaction of aromatic aldehyde and phenylacetic acid yielded an intermediate of carboxyl stilbene [140]. *cis*-Stilbene was then obtained by removing the carboxyl of the intermediate [141]. In addition, condensation of substituted aniline and benzaldehyde will easily generate Schiff-base type stilbenes [142] (Scheme 15e).

### Total-synthesis strategies of chalcone derivatives

Chalcones not only exist extensively in plant as the precursor of flavonoid, but also can be easily obtained by various total-synthesis strategies. Several chalcone derivatives have already been developed as clinical drugs such as Sofalcone and Metochalcone [14]. The most classical total-synthesis strategy of chalcone fragments is aldol condensation of substituted acetophenone and benzaldehyde in the presence of alkali, which might cause the disproportionation (Cannizzaro reaction) of benzaldehyde and secondary reaction of Michael addition (Scheme 16a) [143, 144]. For improving the selectivity and yield of aldol condensation, current researches mainly focus on novel reaction conditions such as microwave [145] and ultrasound [146] and developing novel catalysts. Traditional basic catalysts were replaced by organic base [147] and solid superbase [148] which realized higher selectivity and yield. A variety of acid catalysts including hydrochloric acid [149], *para*-toluenesulfonic acid [150] and Lewis acids [151, 152] were also applied, especially suitable for the aromatic aldehydes with phenolic hydroxyls. Chalcones can also be produced through cross-coupling reaction of benzoic or cinnamate derivatives with organoboron or organometallic compounds mainly including arylboronic acids, aryllithium and arylstannanes catalyzed by transition metal catalysts [153–155] (Scheme 16b). Compared with the good performance and wide application of organoboron, organometallic compounds were rarely used anymore as the reactants of chalcone. Friedel–Crafts acylation of substituted benzene with cinnamoyl chloride derivatives is also a common strategy for total-synthesis of chalcone fragments using protonic or Lewis acid as the catalyst [156] (Scheme 16c). By the same way dihydrochalcones can be provided from phenylpropionyl chloride. Dehydrogenation of diarylpropenols is another strategy to generate chalcone derivatives (Scheme 16d). It always occurred in the presence of manganese dioxide [157], pyridinium chlorochromate (PCC) [158] or transition-metal catalysts [159], which was recently replaced by metal-free oxidations such as CO_2_ catalytic system [160], owing to their easy separation, low toxicity and cheaper price. Diarylpropenols, the precursor of chalcones, can be obtained by cross coupling of aldehyde with organolithium [161] or Grignard reagent [162] (Scheme 16d). As another reduction state of chalcone, dihydrochalcone was always obtained by catalytic hydrogenation of chalcone, while it can also be directly synthesized by catalytic alkylation of ketones. Recently, a ligand-free alkylation of ketones with primary alcohols was developed using FeCl_2_ as the catalyst (Scheme 16e), exhibiting a wide application prospect in rapid synthesis of dihydrochalcones [163].

### Total-synthesis strategies of flavonoid derivatives

Flavonoids are a large class of polyphenol widely distributed in natural vegetation with a C6–C3–C6 backbone. Depending on the structures, flavonoids can be categorized as flavone, flavanone (dihydroflavone), flavonol, isoflavone, aurone and so on. Cyclization of 2'-hydroxychalcones was a universal strategy to synthesize different types of flavonoids (Scheme 17). Amongst them, flavanone could be easily obtained by cyclization of 2'-hydroxychalcones under the catalysis of alkali or acid. [164, 165] Oxidation of flavanone by IBX/DMSO system will generate flavones, while catalytic hydrogenation could transform flavones back to flavanone. Flavones can also be produced by oxidative cyclization of 2'-hydroxychalcones, occurring in the presence of iodine with the solvent of dimethyl sulfoxide [166, 167]. Triethylene Glycol (TEG) was a common alternative solvent, might leading to higher yield for specific flavones [168, 169]. However, excess iodine and reaction time will give rise to the formation of 3-iodoflavones [170], while novel iodine sources such as iodine monochloride [171] and ammonium iodide [172] have been developed to solve this problem. In a water-mediated phosphorylative cyclodehydrogenation, flavones and flavanones can be synthesized in the same condition with the product type controlled by dosage of reagent [173]. As the oxidative state of flavones, flavonols can be produced from flavones by the oxidation with 3,3-dimethyldioxirane (DMDO) [174] or Algar-Flynn-Oyamada reaction in the presence of base and hydrogen peroxide [175, 176], which may generate by-product of aurone due to the different attacking position of epoxide [177]. Selective synthesis of aurone could be achieved through oxidative cyclization of 2'-hydroxychalcones mediated by Hg(OAc)_2_ in a good yield [178, 179]. Aurones can also be converted to flavonols by bromination, elimination and rearrangement, which was known as Auwers synthesis [180].

Classical synthetic methods of flavonoids were limited by the preparation of 2'-hydroxychalcones. Nowadays, novel and rapid synthetic strategies of flavonoids have been developed and gradually replaced the classical methods. Two-step synthesis from 2-hydroxyacetone and benzoyl chloride has been the most common strategy to generate flavones (Scheme 18a) due to its excellent applicability and convenience [181–183]. Firstly, β-propanedione were synthesized from 2-hydroxyacetone and benzoyl chloride via esterification and base-mediated Baker-Venkataraman rearrangement. Secondly, cyclization of β-propanedione generated flavones. Addition of phase transfer catalysts such as tetrabutylammonium hydrosulfate could make 2 steps in one-pot [184]. It’s also reported flavones were synthesized directly from β-ketoester and phenol by microwave irradiation under eco-friendly solvent-free conditions in a high yield [185] (Scheme 18b). Under dual catalysis of FeCl_3_ and piperidine, flavones could also be directly supplied from salicylaldehyde derivatives and substituted phenylacetylenes with atmospheric oxygen as the stoichiometric oxidant [186] (Scheme 18c). As the reduction state of flavones, flavanones could be directly synthesized from 2-hydroxyacetophenone and benzaldehyde by the catalysis of aniline via Mannich-type reaction [187, 188] (Scheme 18d). In addition to the cyclization of 2'-hydroxychalcones which is presented in Scheme 17, aurone and its derivatives can be easily synthesized by Claisen-Schmidt reaction of aromatic aldehyde with benzofuranone derivative [189] (Scheme 18e). Over the last decade, a novel approach was developed to efficiently prepare flavones and aurones from *o*-alkynoylphenols (Scheme 18f), while the product types were determined by catalytic system. Employing trifluoroacetic acid or dimethylaminopyridine as the catalyst regioselectively promoted the generation of flavones [190]. Using tributylphosphine [191] or alkali salt such as cesium carbonate [192] mainly produced aurones. As the intermediate, *o*-alkynoylphenols can be synthesized by cross-coupling of salicylaldehyde derivatives with alkynyl benzene [193]. Similarly, flavones and aurones can be synthesized from *o*-hydroxylpropinyl phenols, the reduzate of *o*-alkynoylphenols, through stoichiometric oxidation and catalytic cycliczation [194, 195].

Isoflavones were always directly synthesized by Vilsmeier-Haack reaction of deoxybenzoin with formyl equivalent such as methylsulfonyl chloride and DMF [196, 197] (Scheme 19a). Base catalyzed condensation of deoxybenzoin with paraformaldehyde will provide isoflavanone [119], which can be promoted by Microwave [198]. The deoxybenzoin intermediate was always supplied by a Friedel–Crafts acylation of phenol and phenylacetic acid derivatives. Isoflavones can also be synthesized by a three-step strategy (Scheme 19b). Firstly, the intermediate was synthesized through the condensation of *o*-hydroxy acetophenone with DMF. Then it was cyclized under the action of iodine to generate 3-iodochromone [199]. Isoflavones were finally obtained by the cross-coupling of 3-iodochromones with arylboronic acids [200].

### Total-synthesis strategies of complex natural phenol derivatives

Total-synthesis of complex natural phenols was still a challenge due to the regioselectivity of multi-reactive sites and the enantioselectivity of multi-chiral centers, especially for non-symmetrical polyphenols. For symmetric natural polyphenols (i.e., the polymers of simple phenols), the general total-synthesis strategy can be summarized as synthesis of simple phenol and then coupling. Chemo- and regio-selective reactions including the protection/deprotection of hydroxyl groups were required which have been discussed above. As a solution to control the stereochemistry, the configuration of alcohol hydroxyl groups can be reversed by dehydrogenation and selective reduction. For instance, dehydrogenation with Dess-Martin periodinane (DMP) followed by reduction with L-selectride provided exclusively the cis-substituted talienbisflavan A [201] (Scheme 20).

Besides the coupling of simple phenol by methylene in above example, symmetric natural polyphenols were always directly coupled by regioselective oxidation. In the biosynthetic process of gossypol, a complicated natural polyphenol, it was dimerized from hemigossypol by peroxidase in the presence of dirigent protein that controlled the enantiomeric ratio of product [202] (Scheme 21). Inspired by the biosynthetic process, hemigossypol was also pre-synthesized in the artificial total-synthetic process of gossypol, and then coupled by an enzyme-free peroxide system [203]. Although the coupling strategies of phenol units can be referenced, the preparations of phenol units are totally individualized for different products. The construction of C-O bond on benzene ring with high selectivity and efficiency was the key challenge.

## Conclusions and perspectives

As described in this review, a great abundance of strategies can be applied in the derivatization of natural phenols, including semi-synthesis strategies based on common fragments and total-synthesis schemes of typical natural phenols. In the section of semi-synthesis, almost all classical strategies have been covered, which are preferred in initial trial period as they can rapidly construct the molecular library of derivatives. Those regioselective strategies are particularly suited for complex natural phenols with multiple reactive centers, while diastereoselective strategies could promote the generation of single enantiomer products as the conformation have a significant influence on biological activity. Total-synthesis strategies are suggested for natural phenols with rare resources. More importantly, they can avoid the limitation of original skeleton, which provide the possibility for diversity-oriented derivatization.

Unlike previous reviews analyzing how to design bioactive structures, this review focuses on providing available options of structural derivatization as diverse as possible, which will mediately help inspire the ideas of structure design. Researchers still need to select from these options according to their interests combined with drug design theory and technology. Due to the great variety of chemical reaction conditions and the limitation of space, only the common conditions were listed in this review. As referred in the review, some reactions have already been discussed systematically and separately in previous reports. In addition, it’s still a challenge to formulate total-synthesis schemes for novel and complex natural phenols, as there is no unified total-synthesis strategy for different types of natural phenols. Researchers also need consider whether there are reactive groups requiring protection before applying these strategies, while selective protection and deprotection strategies for phenolic hydroxyls have been discussed in the review.

Natural products play an irreplaceable role in drug research and development, as they are structurally complementary to synthetic compounds. However, with the decrease of newly discovered natural compounds, especially from land surface, the research hotspot has gradually transformed from traditional discovery and isolation into in-depth and secondary development. In the meantime, the emergence and development of high-throughput-screening technologies have significantly improved the efficiency of bioactivity screening, leading to greater demand of the derivative quantity and diversity. Structural derivatization is one of the most important way to continuously develop the potentialities of existing natural compounds, and meanwhile provide more samples for high-throughput-screening. Description in this review will help to take full advantage of classical and advanced strategies, break the barrier between the fields of natural phenols and chemical synthesis, and inspire the design of derivative structures. Currently, increasing organic reactions and biocatalytic reactions were developed [204], as well as synthetic biology which can realize the bio-derivatization [205]. Application of novel techniques will supply more chances and diversity for structural derivatization of natural phenols.
